# Alternative Ion-Pairing Modifiers Should Be Investigated in Low-Input and Single-Cell Proteomics

**DOI:** 10.1021/acs.jproteome.5c00930

**Published:** 2025-11-24

**Authors:** Colten D. Eberhard, Cameron Braswell, Benjamin C. Orsburn

**Affiliations:** † The Department of Pharmacology and Molecular Sciences The Johns Hopkins University School of Medicine,Baltimore, Maryland 21205, United States; ‡ Organ Pathobiology and Therapeutics Institute University of Pittsburgh, Pittsburgh, Pennsylvania 15203, United States

**Keywords:** single-cell proteomics, diaPASEF, DIA, alternative buffers

## Abstract

A recent study demonstrated a substantial increase in the peptide signal and corresponding proteome coverage when employing 0.5% acetic acid (AA) as the ion pairing modifier in place of the 0.1% formic acid traditionally used in shotgun proteomics. Given the strictly limited material and counterintuitive observations by others in the emerging field of single-cell proteomics, we chose to investigate this alternative modifier in the analysis of subnanogram proteome dilutions. When digest standards as low as 20 pg total load on the column were evaluated, AA led to increased proteome coverage at every peptide load assessed. Relative improvements were more apparent at lower concentrations, with a 20 pg peptide digest demonstrating a striking 1.8-fold increase to over 2000 protein groups identified in a 30 min analysis. Furthermore, we find that this increase in signal can be leveraged to reduce ramp times, leading to 1.7× more scans across each peak and improvements in quantification, as measured by %CVs. These results can be reproduced on multiple trapped ion mobility instruments. When evaluating single cancer cells, approximately 13% more peptide groups were identified on average when employing AA in the place of FA. These results suggest that ion pairing modifiers and other additives warrant re-evaluation in the context of low-input and single-cell proteomics. All vendor raw and processed data are available through ProteomeXchange as PXD046002 and PXD051590.

## Graphical abstract

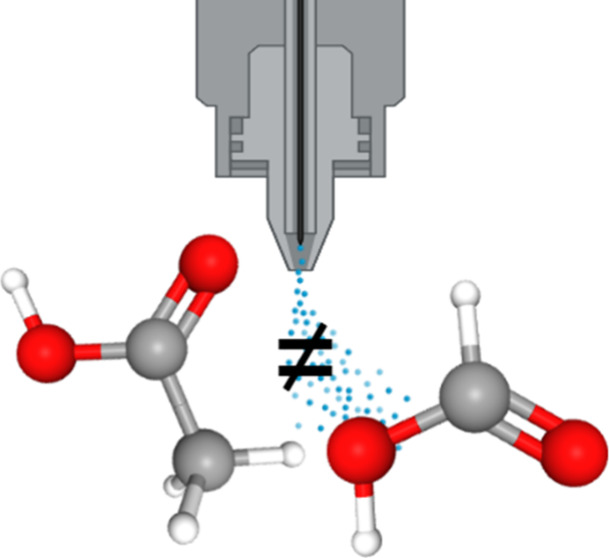

## Introduction

Single-cell proteomics (SCP) by liquid chromatography–mass spectrometry (LCMS) is a growing field of research driven by rapid innovation in sample preparation,^1,2^ chromatography,^3^ mass spectrometry methods,^4–6^ instrumentation,^7^ and data analysis.^8–10^ Despite these advances, improvements in peptide detection efficiency are still essential, as it has become a critical parameter when working with a finite amount of material in an extremely complex form. Recently, Battellino^11^ demonstrated that substituting 0.1% formic acid (FA) for 0.5% acetic acid (AA) in their LCMS proteomics workflow increased peptide signal 2.5-fold, with only slight decreases in chromatographic resolution.^11^ While the use of 0.5% AA may provide negligible benefits in shotgun proteomics workflows, where the sample is typically less sparse, this simple change of acid could result in a remarkable improvement in SCP and workflows. If true, this may open up other additives that should be considered.

## Methods

### Preparation of K562 Dilution Series

Promega human cancer cell line tryptic digest (V7461) was serially diluted using a solution containing 0.1% FA in LCMS grade water (Pierce 85170) and 0.1% n-dodecyl-beta-maltoside detergent (DDM, Thermo Fisher, 89902). A total of 10 μL of each dilution, 10, 100, and 1000 pg/μL, were loaded into 16 wells each of a 96-well plate with 100 μL total volume per well (Thermo 60180P210). The plate was tightly sealed with plate sealing adhesive tape (Fisher, 60180-M143) and centrifuged prior to loading onto the autosampler.

### Isolation and Preparation of Cancer Cells

Cancer cell lines were obtained from ATCC and grown in the appropriate culture media described by the vendor and, according to bank policies, were used with proper ethics. PANC 0203 cells were grown in RPMI 1640 (ATCC 30–2001) supplemented with 15% fetal bovine serum (FBS) (ATCC 30–2020) and 10 units of human insulin (Fisher). SW620 cells were grown in Leibovitz’s L-15 Medium (ATCC 30–2008) supplemented with 10% FBS (ATCC 30–2020). NCI-H-358 cells were grown in RPMI 1640 (ATCC 30–2001) supplemented with 10% FBS (ATCC 30–2020). All culture media were supplemented with 10 mg/mL Penn Strep antibiotic solution (ATCC 30–2300). All cell lines were passaged a minimum of 3 times prior to single-cell isolation. Cells were harvested first by vacuum aspiration of the cell culture media. The adherent cells were briefly rinsed in 3 mL of a 0.05% trypsin plus EDTA solution (ATCC 30–2001). This solution was rapidly aspirated off and replaced with 3 mL of the same solution. The cells were examined by light field microscopy and incubated at 37 °C with multiple examinations until the adherent cells had lifted off the plate surface, approximately 5 min. The active trypsin was then quenched by the addition of 7 mL of the original culture media. The 10 mL solution was transferred to sterile 15 mL Falcon tubes (Fisher) and centrifuged at 300 × *g* for 3 min to pellet the cells. The supernatant was gently aspirated off, and the cells were resuspended in PBS solution without calcium or magnesium with 0.1% BSA (both, Fisher Scientific) at a concentration of 1 million cells per mL, as estimated by bright field microscopy. Cells for single-cell aliquoting were gently dissociated from clumps by slowly pipetting a solution of approximately 1 million cells through a Falcon cell strainer (Fisher, 353420). The cells were placed on wet ice and immediately transported to the Johns Hopkins University Bloomberg Flow Cytometry and Immunology Core. Nonviable cells were labeled with a propidium iodide solution. The cell suspension was briefly vortexed prior to cell isolation and aliquoting.

Single cells were aliquoted using an analogue MoFlo sorter into cold 96-well plates containing 2 μL of LCMS grade acetonitrile (one cell per well). Following aliquoting, each plate was immediately sealed and placed in an insulated box of dry ice, with the wells pressed into the material to ensure rapid cooling. The plates containing frozen single cells were stored at −80 °C (PANC 0203 and H-358) or processed immediately (SW620). For processing, acetonitrile was driven off by heating the cells for 90 s at 95 °C using a 96-well hot plate. Dried cell lysates were digested using 2 μL of a digestion solution containing 5 ng/μL LCMS grade trypsin (Pierce) in 0.1% DDM (Thermo Fisher, 89902) and 50 mM TEAB (Thermo Scientific). The plates were sealed with adhesive plate tape (Fisher, 60180-M143), and digestion occurred at room temperature overnight. Following digestion, the plates were briefly centrifuged to condense evaporation, and wells were completely dried under vacuum centrifugation (Eppendorf Vacufuge Plus). Resulting peptides were resuspended in 3.5 μL of 0.1% FA, vortexed, and centrifuged prior to loading on the autosampler.

### LCMS Instrument Parameters on TIMSTOF SCP

An EasyNLC 1200 system (Proxeon) coupled to a TIMSTOF SCP instrument (Bruker Daltronics) was used for initial samples and for all isolated single human cells. Peptides were separated using a constant flow of 300 nL/min on an IonOpticks 15 cm × 75 μm C-18 column with 1.7 μm particle size (Ion Opticks “Ultimate”) with an integrated CaptiveSpray emitter held at 1500 V. For standard injections, an appropriate volume (1–4 μL) of each well was loaded with a partial loop injection of 7–10 μL at 900 bar. Pickup and partial loop injection volume values (2 × pickup volume +2 μL if >7 μL) were used according to the recommendations of the “How to set up the EasyNLC method” document by the University of Washington Proteome Research Center (https://proteomicsresource.washington.edu/). For single-cell injections, 4 μL was picked up with a partial loop injection volume of 10 μL. Mobile phase A consisted of either 0.1% FA (Thermo Scientific) or 0.5% AA (Fisher Scientific) in water (Fisher Chemicals). Mobile phase B consisted of 80% acetonitrile in water with the appropriate ion pairing modifier for each experiment. All reagents were LCMS grade. The 30 min gradient used in all experiments began at 8% B and ramped to 35% B by 22 min. The gradient then increased to 100% B by 26 min, where it held for 2 min before returning to baseline conditions. The column was equilibrated in 12 μL of baseline conditions prior to each injection.

The TIMSTOF SCP system was operated in diaPASEF mode using a method with 50 Da isolation windows provided by Michael Krawitzky of Bruker Daltronics during instrument training and will be referenced as the “default parameters” in the study. In this method, the MS1 scanned from 100 to 1700 *m*/*z* with a 1/k0 window of 0.6–1.4. Ions that entered the mass analyzer were restricted by a user defined polygon set on the *m*/*z* vs 1/k0 heatmap. The polygon can impart dramatic alterations on the ion signal and thus must be carefully optimized for each series of experiments. In this experiment, the polygon began at 300 *m*/*z* and was set to not acquire ions in the MS1 cloud below 800 *m*/*z*. The method utilized 6 cycles with 3 isolation windows per cycle, resulting in a total time of 1.2 s when a 166 ms ramp time was used. The “high sensitivity” mode was enabled for all of the samples. For the 100 ms experiments described, the ramp time was decreased from 166 to 100 ms to allow a shorter cycle time of 0.72 s. To prepare the instrument for samples utilizing a different ion pairing modifier, mobile phase bottles were changed, and 5 full purge cycles were performed. The precolumn and analytical columns were equilibrated using 10 and 12 μL of mobile phase A, respectively, at 900 bar to flush the previous ion pairing modifier from the columns.

### LCMS Instrument Parameters on TIMSTOF Ultra2

An EvoSep One HPLC coupled to a TIMSTOF Ultra2 system was used as a second system for this study using the vendor supplied “80SPD Whisper Zoom” method, which features a gradient and run-to-run time of approximately 16.5 min. The sole alteration to this method is the use of a 10 cm PepSep column (10 cm × 75 μm with 1.9 μm Reprosil), which outperforms the 5 cm column recommended by the vendor in every analysis performed on this instrument (data to be published elsewhere). Diluted digests of the K562 standard were prepared in 0.1% FA in LCMS-grade water containing 0.015% DDM solution. All other EvoTip loading and operation procedures were performed as described in vendor inserts. The TIMSTOF Ultra 2 system utilized a vendor default “short gradient high sensitivity” method for diaPASEF. Briefly, the 1/k0 limits were set at 0.64 to 1.50, respectively. Within a total cycle time of 0.96 s, a single MS1 ramp and 8 MS/MS ramps with 24 windows were analyzed. The 24 windows covered an MS/MS mass range from 400 to 1000 *m*/*z* in 25 Da windows and 0.64 to 1.37 1/k0 values. For mobile phase comparisons, FA and AA solutions were prepared identically, and a full system solvent exchange protocol was performed between each injection.

### Data Analysis

Prior to analysis, all raw files were converted to the HTRMS file format using the “HTRMS Converter” software (Biognosys) using the default parameters. These files were then analyzed in Spectronaut 18 (Biognosys) via the directDIA+ workflow with default parameters for data calibration and analysis. The UniProt SwissProt reviewed library for human (downloaded on March 3, 2023) and appended with the cRAP contaminant database (www.gpm.org) was used for library generation. For standard digests, carbamidomethylation of cysteines was considered as static, and methionine oxidation was considered a dynamic modification. Single cells were analyzed exclusively by methionine oxidation. Output data was visualized in GraphPad Prism 10.0.1. All Bruker.d raw files and Spectronaut processed results have been made publicly available through the ProteomeXchange partner repositories^12^ as PXD046002 and PXD051590.

## Results

### AA Improves Proteomic Coverage on Subnanogram Injections of Peptides

To determine whether the use of FA or AA in the mobile phase impacts proteomic coverage, a dilution series of a standard trypsin digest was injected into a TIMSTOF SCP system with 20, 100, 200, 400, and 1000 pg total peptide on the column using both mobile phase conditions. Our data show an increase in detected precursors, peptides, proteins, and protein groups in the 20–200 pg dilutions. Interestingly, as peptide concentration on the column increased from 20 to 200 pg, the number of additional identifications gained by the AA modifier compared to the FA modifier decreased. Remarkably, at 20 pg of digest standard, the number of unique peptides detected nearly doubled, leading to the striking observation of 2,000 protein groups at this concentration (Figure 1A/B). Of note, at 200 picograms or above of standard trypsin digest on the column, the number of precursors and peptide groups was higher in the AA but did not translate to a significant increase in protein groups detected (Supporting File 1). In a more limited dilution series on a TIMSTOF Ultra2 system equipped with an EvoSep One system and a shorter overall LC gradient (45 min compared to 16 min), the same trends are observed (Figure 1C/D).

**Figure 1:**
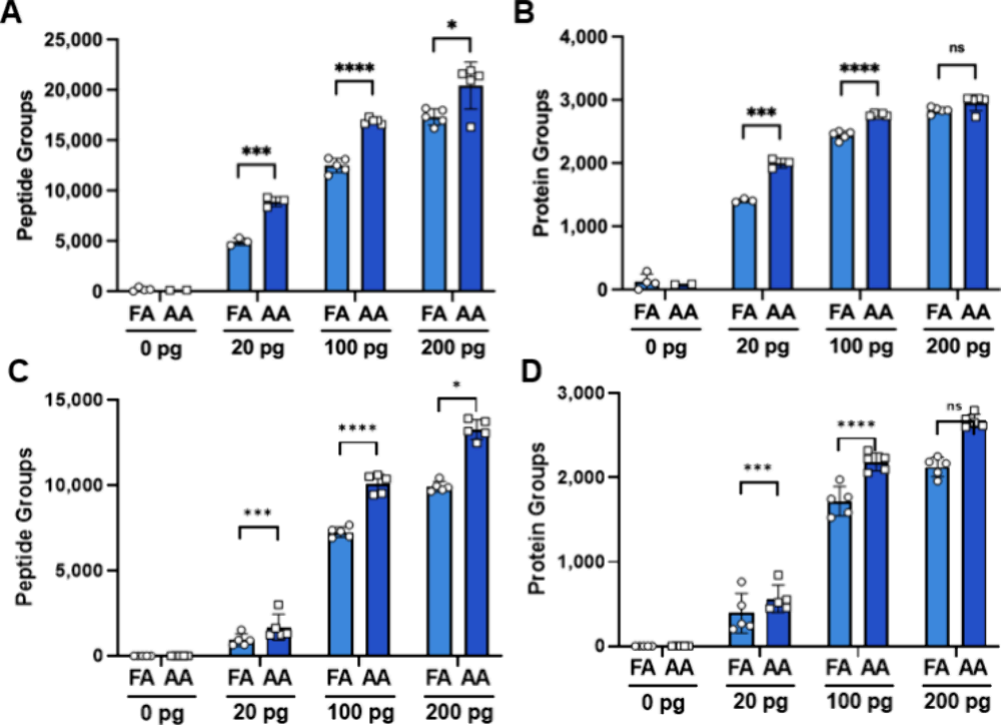
Proteomic coverage of subnanogram injections of a standard trypsin digest. (A) Peptide and (B) protein groups identified at 20, 100, and 200 pg of peptide digest with 0.1% formic acid (FA) or 0.5% acetic acid (AA) in the mobile phases. (C) Peptide and (D) protein groups of the same dilutions and conditions on a TIMSTOF Ultra2 with an EvoSep One system with 16.5 min run to run analysis time. Error bars represent mean and standard deviation. Statistical analyses were performed using a two-tailed *t-*test. *P*-value <0.05*, <0.001 ***, <0.0001****; ns, no significance.

### Increased Relative Signal from AA Can Be Used to Obtain More Scans across Each Peak

In addition to an increase in the number of peptide and protein groups identified, the overall signal intensity was also higher when AA was used as the ion pairing modifier. We hypothesized that the increased relative signal provided by AA could be exploited to reduce the relative ion accumulation time, resulting in more scans across each peak. The default ramp time on the TIMSTOF SCP system and vendor provided methods is 166 ms, resulting in a cycle time of 1.2 s. If the ramp time is decreased to 100 ms, the instrument software estimates a cycle time of 0.7 s, allowing 1.7 times more scans across each peak. In general, more measurements allow improved relative quantification, which is typically assessed in shotgun proteomics by calculating the coefficient of variation (%CV).^13^ We next repeated the analysis of the digest dilution series using the 100 ms ramp time with the AA mobile phase system. The resulting number of peptides with %CVs below 20 and 10% was then extracted from the Spectronaut postanalysis report (Figure 2). We observed that in the 100 and 200 pg standard trypsin digests the number of peptides with low CVs increased with the substitution of AA and, further, with the decrease in ramp time. However, the 20 pg dilution in the AA mobile phase with a 100 ms ramp time exhibited a decrease in the quantitative accuracy when compared to the 166 ms ramp time.

**Figure 2:**
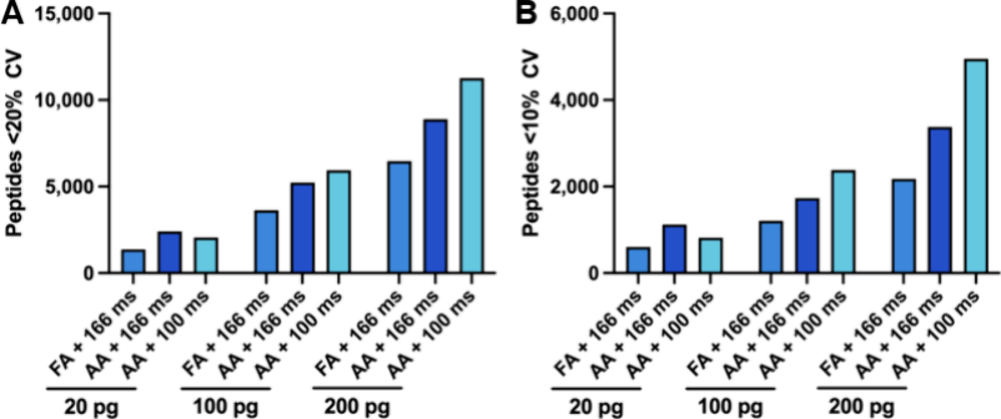
Number of peptides quantified with low %CVs. Peptides quantified at less than (A) 20% CV and (B) 10% CV when injecting 20, 100, and 200 pg of standard trypsin digests with 0.1% formic acid (FA) or 0.5% acetic acid (AA) with ramp times of 166 or 100 ms.

### AA Increases Protein Sequence Coverage in Isolated Single Human Cells on a TIMSTOF SCP System

Encouraged by these results, we repeated this analysis using isolated single human cancer cells from three separate cell lines. Remarkably, using this extremely simple single-cell preparation method, over 800 protein groups were identified on average across the 98 single cells that returned quantifiable data (Supporting File 1). In evaluating the impact of the ion pairing modifier on SCP, we observed a general upward trend in both peptide and protein groups identified when employing AA (Figure 3, Supplemental Figure 1). Of particular interest, single H-358 cells were analyzed with both the default 166 ms and reduced 100 ms ramp time methods. When utilizing a ramp time of 166 ms, an improvement of approximately 14% was observed in the number of peptides and precursors identified for single cells run using AA solvents (Figure 3A). It should be noted that a two-tailed *t* test still found this increase to be significant even when the two lowest cells analyzed with FA solvents were removed from consideration (data not shown). However, the increase in the number of peptides did not lead to a significant increase in the number of protein groups identified (Figure 3B). When the 100 ms ramp time was utilized, a higher number of peptides and proteins were observed when AA was used as a modifier, but in neither case was this improvement found to be statistically significant. Similar to the 20 pg peptide standard, the number of peptide and protein groups was lower when the 100 ms ramp time was used.

**Figure 3:**
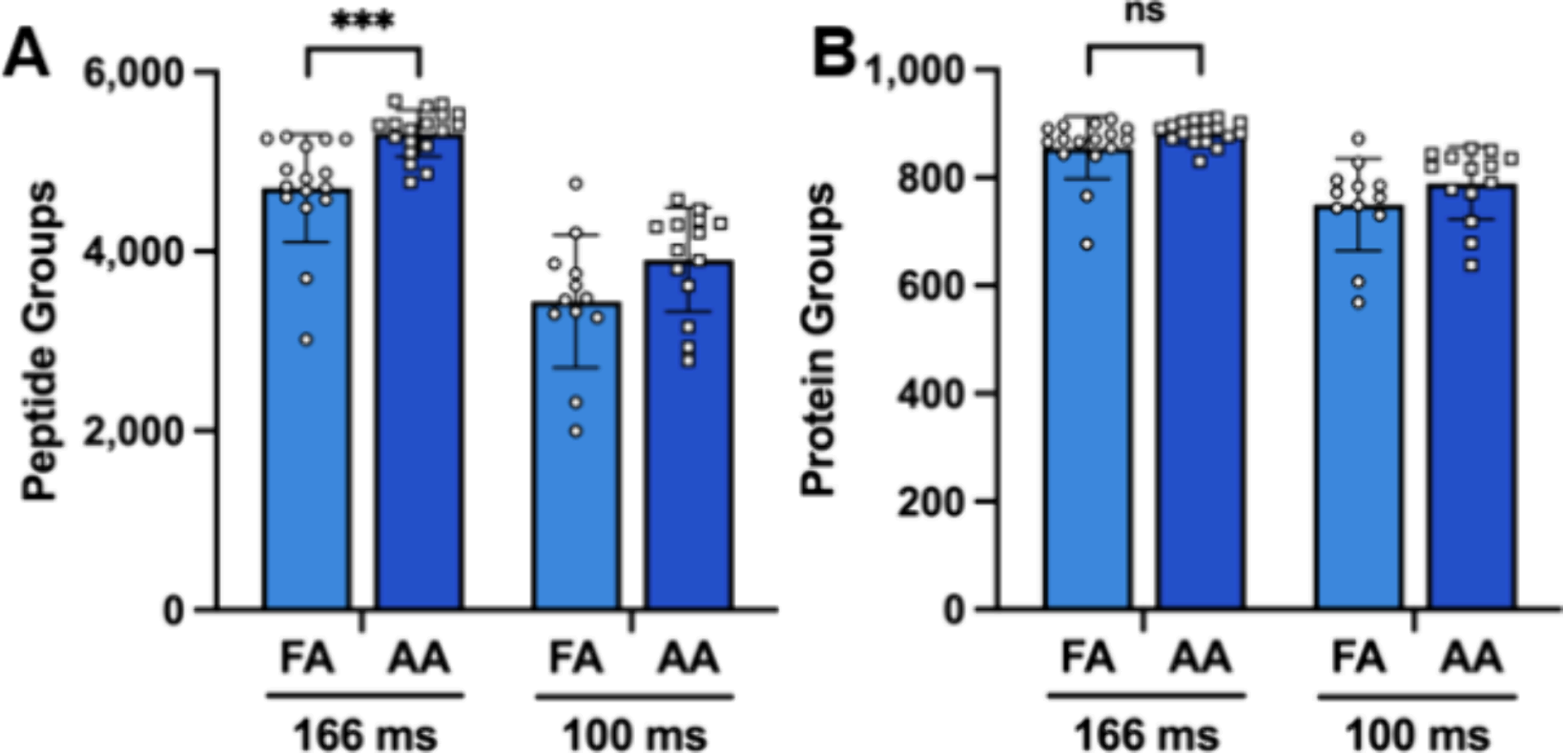
Peptide and protein groups identified in single cells. (A) Peptide and (B) protein groups identified in single H-358 cancer cells with 0.1% formic acid (FA) or 0.5% acetic acid (AA) in the mobile phases when employing ramp times of 166 and 100 ms. Error bars represent mean and standard deviation. Statistical analyses were performed using a two-tailed *t-*test. *P*-value <0.001 ***, ns, no significance.

## Conclusions

Incorporating additives to increase the peptide signal and identification rates is not a new concept. The addition of 5% DMSO has long been noted as an additive that can lead to impressive increases in peptide and protein coverage relative to 0.1% FA alone.^14^ As noted in Battellino et al., however, less than 2% of all proteomics studies in the literature have employed DMSO. We suspect this is due to reports of contamination issues after long-term use, which can require extensive cleaning of internal instrument components. Also detailed in the article, this study is based upon is the interesting historical move of early proteomics laboratories from AA to FA, which the authors suspect was due to the challenges of multidimensional online chromatography techniques.^11^ AA appears to provide comparable increases in peptide signal to DMSO and has been employed in LCMS systems used for the analysis of extractables and leachables and pesticides for decades.^15,16^ It seems unlikely, for this reason, to be detrimental to long-term instrument performance. With similar increases in the total ion signal, AA is an attractive alternative and appears applicable across a wide range of gradients and instrument conditions. In Battellino,^11^ gradients between 60 and 120 min in length were effective for proteomics on both QTOF and Orbitrap analyzers. Here we have found similar results for 30 min gradients on TIMSTOF systems designed for low input samples. This improvement in signal may be attributed to the lower Ka of AA compared to FA, which may be a detriment in higher concentration samples but an asset as concentration decreases, a hypothesis that we thank Dr. Ryan Kelly for sharing. Following the initial preprint of this manuscript, the principal investigator moved and established a new laboratory with more recently released instrumentation. While using a closely related TIMSTOF Ultra2, but a dissimilar HPLC in the EvoSep One system, we find these data again reproduce. Our standard workflow utilizes a much shorter gradient and run-to-run acquisition time on this new hardware (approximately 16 min run-to-run, compared to 45 min) with similar results. This configuration does appear to have significantly lower numbers of proteins identified in the blanks, likely due to the disposable EvoTip cartridges. Interestingly, though, this workflow appears to have fewer overall peptide numbers, particularly at 20 ng peptide loads, which may be due to loss of transfer of peptides from tube to EvoTip and subsequent washing. New methods for using EvoSep systems for low-input proteomics have resorted to sorting cells directly into the tips or transferring them to tips through gravity to eliminate pipetting losses for these reasons.

While it is curious that we see less of an improvement in coverage when analyzing isolated single human cells, we observe a general trend of improvements in three separate preparations of single cells. The evaluation of this disconnect is outside of the scope of this study but could simply be due to sampling bias, as only 98 cells were successfully analyzed across three separate cell lines and two different instrument methods. We can, however, make some inferences here on the amount of sample loss in this simple one-pot sample preparation method. Work in our group previously estimated each H-358 cell grown in these culture conditions to have an average protein content of 200 pg.^6^ At a peptide group level, we observe approximately the same number of peptides as the 20 pg standard digest, though with lower overall protein numbers. Similarly, we find that decreasing the TIMS ramp time to 100 ms from the default 166 ms method results in a loss in proteome coverage in both single cells and 20 pg of standard digest. Taken together, these results suggest that this single-cell preparation method results in a 90% loss of protein content. This may not be surprising given the relatively large surface area available for peptide adhesion within a standard 96-well plate, compared to loss inferences from cells prepared directly within 384-well plates in recent studies.^17–19^ Our interpretation is that the use of highly diluted peptide standards as a proxy for isolated single cells, while seemingly practical, may produce results that should be interpreted with a high degree of caution. However, the final conclusion of this technical note is that the emerging field of SCP should continue to question traditional proteomics methods at every stage, as remarkable increases in coverage appear to be achievable through relatively minor optimizations.

## Supplementary Material





## Notes

Excel sheet with the number of precursor, unique peptide, and protein group identifications for all K562 serial dilutions and single cells analyzed in this study (XLSX)
Bar plots of peptide and protein groups from additional single cancer cells analyzed with AA and FA solvents (PDF)
