# Supplementary material for: Alternative Ion-Pairing Modifiers Should Be Investigated in Low-Input and Single-Cell Proteomics
Source: J Proteome Res. 2025 Nov 24;24(12):6338–43. doi: 10.1021/acs.jproteome.5c00930 (PMC12687309; doi:10.1021/acs.jproteome.5c00930)

Supporting Information for

Alternative ion-pairing modifiers should be investigated in low-input and single cell proteomics

Colten D. Eberhard[1], Cameron Braswell[2] and Benjamin C. Orsburn[2*]

[1]The Department of Pharmacology and Molecular Sciences
The Johns Hopkins University School of Medicine, Baltimore, MD, USA, 21205
[2]Organ Pathobiology and Therapeutics Institute
University of Pittsburgh, Pittsburgh, PA, US, 15203
[#]Corresponding: orsburn@pitt.edu

Table of contents:

Figure S1. Bar plots of peptide and protein groups from additional single cancer cells analyzed with AA and FA solvents.

File S1. An Excel sheet with the number of precursor, unique peptide, and protein group identifications for all K562 serial dilutions and single cells analyzed in this study. (xlsx)

Supporting Figure S1. Peptides and proteins obtained from single cells. (A) Peptide and **(B)** protein groups identified in SW620 and PANC 0203 cells analyzed using a single step digestion method and run using 0.1% formic acid (FA) or 0.5% acetic acid (AA) mobile phase additives. Error bars represent mean and standard deviation. Statistical analyses were performed using a two-tailed t-test and FA vs AA comparisons resulted in no significant differences.

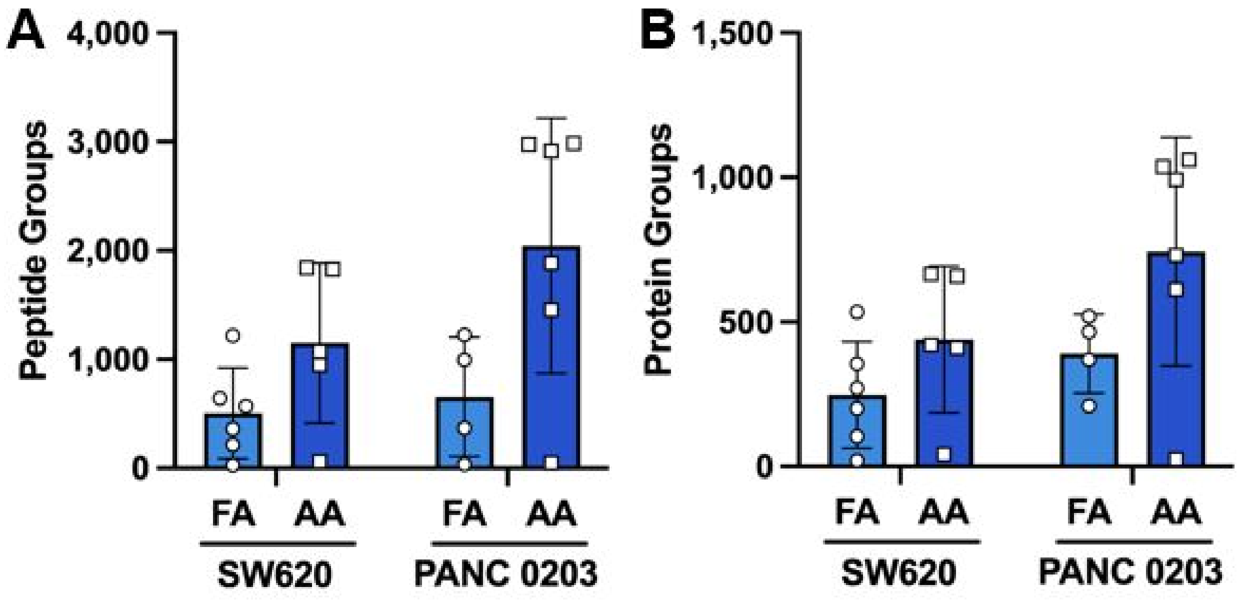

Supplement: Supplementary file 2 [file pr5c00930_si_002.pdf]
